# Evaluating a Telemedical Follow-Up Program for Continuity of Care After Hospital Discharge: Prospective Clinical Intervention Study

**DOI:** 10.2196/85467

**Published:** 2026-03-27

**Authors:** Jasmin Stéphanie Gram, Rebecca Meier, Bianca Hölz, Khaled Abdelhamid, Tobias Lang, Linus Imfeld, Henry Rotte, Iris Warthmann, Simone Zweipfenning, Kerstin Casper, Rebecca Häner, Alina Lara Schödler, William Thai, Claudia Abel, Noémie Aeschlimann, Mona Ingrisani, Isabelle Ledergerber, Carsten Sommer-Meyer, Veronique Mayer, Michael Handke, Joelle Kaufmann, Stéphanie van der Lely, Timo Rimner, Cornelia Gujer, Rahel Ganarin, Alfred Angerer, Sven Hirsch, Susanne Suter, Krisztina Schmitz-Grosz, Jens Eckstein

**Affiliations:** 1 Department of Internal Medicine, University Hospital Basel, Petersgraben 4, Basel, 4031, Switzerland, 41 792759501; 2 Department Digitalization & ICT, University Hospital Basel, Basel, Switzerland; 3 Medgate, Allschwil, Switzerland; 4 School of Computer Science, FHNW University of Applied Sciences and Arts, Windisch, Switzerland; 5 Institute of Health Economics, ZHAW Zurich University of Applied Sciences, Winterthur, Switzerland; 6 ZHAW Research Centre for Computational Health, Institute of Computational Life Sciences (ICLS), ZHAW Zurich University of Applied Sciences, Wädenswil, Switzerland

**Keywords:** hospital at home, transition of care, telemedicine, patient safety, discharge process, telecare, health care environment

## Abstract

**Background:**

The transition from hospital to primary care is a vulnerable period for patients. Telemedicine may enhance continuity of care; however, evidence on its role in supporting hospital-to-primary-care transitions remains limited.

**Objective:**

We implemented and evaluated a telephone-based follow-up program to support the transition from hospital to home and primary care, focusing on patient health as well as patient and health care provider satisfaction.

**Methods:**

In this prospective, single-center intervention study (University Hospital Basel, September 2022-December 2024), 234 patients discharged from the emergency unit or internal medicine wards received structured telemedical follow-up for up to 10 days until their first primary care appointment. Primary outcomes were patient health and satisfaction; secondary outcomes were health care provider satisfaction. Data were collected using patient-reported outcome measures, patient-reported experience measures, and provider assessments and analyzed descriptively and analytically (*P*=.05).

**Results:**

Patient-reported outcome measure and patient-reported experience measure scores changed during follow-up, while no deterioration was observed. Health care provider satisfaction varied, with telemedical physicians reporting the highest ratings, hospital physicians intermediate ratings, and general practitioners the lowest, citing challenges in information transfer and perceived added value.

**Conclusions:**

This study outlines both the potential benefits and the practical challenges of implementing a telephone follow-up program after hospital discharge. Variations in physician satisfaction highlight the need for more user-friendly technical infrastructure and clearer role definitions. Future multicenter studies with broader patient samples, usual care controls, and simplified recruitment processes are required to strengthen the feasibility and generalizability of this approach.

## Introduction

### Background

Transition of care from hospital to home represents a critical phase in the continuum of health care, particularly for older patients who are highly vulnerable to medical and nonmedical complications following discharge. Patients frequently face challenges such as limited understanding of their diagnosis, symptoms, and treatment plans. They also experience uncertainty in adapting to their new situation and often lack adequate support for daily activities and mental health [1-4]. Adverse events following hospital discharge remain challenging in primary care management [5-7]. Most of them have been identified as preventable or reducible through appropriate discharge strategies, which are also associated with a reduced need for subsequent acute care services [3,4,8].

Transitions of care between health care institutions have become a central focus of efforts to enhance patient safety and continuity in care [9,10]. Although definitions vary, patient care transition is generally understood as a multidimensional process involving the handover of care between hospitals, home settings, and primary care providers. It addresses both medical and nonmedical needs of patients and their families through appropriate transitional interventions [10]. Recent research has reported positive impacts of transitional interventions, including timely telephone follow-ups [11,12]. Telephone follow-up is considered an effective modality for reinforcing health information, providing education and guidance, managing symptoms, and identifying complications early. It also offers reassurance, improves adherence, and supports both the physical and emotional well-being of patients [13,14]. However, the type and intensity of required transitional interventions depend on patient disabilities, therapeutic requirements, and specific social and geographical contexts [10].

Telemedicine, encompassing a range of electronic information and communication technologies such as voice calls, videoconferencing, messaging platforms, and other digital tools, enables the remote delivery of health care services [15-17]. It offers substantial advantages by facilitating access to care, supporting remote diagnostic and therapeutic interventions, and potentially reducing time and financial burdens for all parties involved [17,18]. In the context of growing resource constraints in health care systems, demand for telemedicine has increased [18], a trend further accelerated globally by the COVID-19 pandemic [19]. Telemedicine has since remained widely used and is increasingly recognized as a safe and effective alternative to traditional in-person care [20-22]. Patient acceptance has generally been positive [21,22], with documented benefits for emotional well-being, physical activity, and quality of life [23]. Nevertheless, research emphasizes that the effectiveness of telemedicine varies according to medical, social, and geographical determinants. A comprehensive understanding of these factors is essential to ensure equitable access to telemedical services, high user satisfaction, and safe, individualized interventions [24].

### Objectives

Telemedicine has shown benefits across various settings, including transitions between health care institutions, and is generally associated with high patient satisfaction. However, research on telemedicine-based interventions that specifically support the transition from hospital to primary care, considering patient and provider acceptance, remains limited. To address this gap, we implemented and evaluated a telephone follow-up program, focusing on patient health as well as patient and health care provider satisfaction during the transition from hospital to home and primary care. The study was conducted within the framework of the Innosuisse Flagship Project SHIFT (Smart Hospital: Integrated Framework, Tools, and Solutions).

## Methods

### Study Design

This investigator-initiated, prospective, single-center clinical intervention study was conducted at the University Hospital Basel (USB), Switzerland, between September 2022 and December 2024.

### Participants and Recruitment Procedure

Patients were screened for eligibility using predefined inclusion and exclusion criteria applied to electronic medical records in the hospital information system. Patients were eligible if they met all of the inclusion criteria and were excluded if they met any of the exclusion criteria (Textbox 1).

If all eligibility criteria were met, the responsible hospital physician assessed suitability for telemedical follow-up. Subsequently, the patient’s general practitioner (GP) was contacted to ensure collaboration in primary care. Written informed consent was obtained from all participants prior to enrollment.

Textbox 1. **Inclusion and exclusion criteria.**

**Inclusion criteria**

- Adults (18 years and older)
- Hospitalization in the emergency unit or internal medicine ward with a planned discharge within 2 days
- Ability to communicate via telephone and email
- Absence of significant cognitive, psychiatric, or language-related barriers
- Coverage by Swiss Health insurance
- Clinical need for short-term follow-up care suitable for telemedical management

**Exclusion criteria**

- Requirement for in-person follow-up (eg, diagnostic or therapeutic procedures necessitating direct contact)
- Planned transfer to another hospital for further diagnostic or therapeutic interventions
- Indication for inpatient rehabilitation

### Intervention: Telemedical Follow-Up Program

The telemedical follow-up program was delivered by Medgate, a telemedical care provider offering 24/7 physician consultations via telephone and video [25]. On the day of hospital discharge, the telemedical team received a standardized, Health Insurance Network–secured email that included (1) patient identifiers, (2) discharge information (date and location), (3) procedures defined by discharging physician, (4) insurance data, (5) patients contact details (telephone number and email address), (6) GP information (address, telephone number, and email address), (7) most recent laboratory results, and (8) provisional discharge letter.

**Figure 1.**
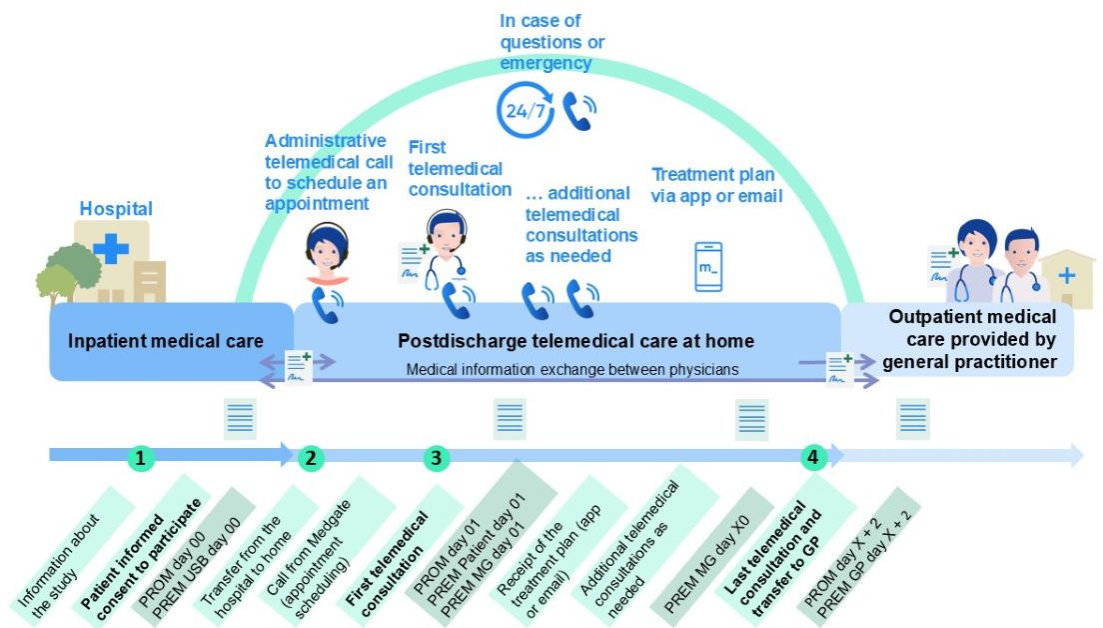
Overview of the telemedical follow-up program, illustrating key contact points and corresponding time points for questionnaire-based data collection. GP: general practitioner; MG: medication guidance; guidance; PREM: patient-reported experience measure; PROM: patient-reported outcome measure.

Patients were contacted by telephone the same day by administrative staff to schedule the first teleconsultation with a telemedical physician (TP) within 24 hours of discharge. Subsequent follow-up telephone calls were scheduled according to the patient’s individual health status and needs. Per protocol, the program was limited to a maximum of 10 days. At that point, care was formally handed over to the patient’s GP or another primary care provider if no GP was available (Figure 1). In the event of adverse outcomes during follow-up (eg, health deterioration and failed contact attempts), the TP assessed the need for rehospitalization. If repeated contact attempts were unsuccessful, the sponsor-investigator was notified and assumed responsibility for further safety measures, including contacting the patient directly and informing the GP if required.

### Data Collection Procedure and Baseline

Patient characteristics, including demographics and medical history, were extracted from electronic medical records of the hospital information system and supplemented by personal interviews, with final documentation in a case report form. Collected data included year of birth, age, sex, height, weight, contact details, GP information, medical history, living and family situation, and the outcome-based nursing assessment for acute care (ePA-AC). The ePA-AC is a daily assessment tool used at the USB that supports the nursing process and classifies activities of daily living. Its numeric outcome, the Self-Care Performance Index (SPI), is derived from 10 ePA-AC questions and reflects the patient’s level of independence in domains such as mobility, dressing, and information processing. Each item is rated on a 4-point scale, producing an SPI score ranging from 0 to 40, with lower values indicating greater care needs [26]. Patients were screened daily by internal ward nurses. Diagnosis clusters were defined by the principal diagnosis most accurately describing the reason for hospitalization.

### Patient Health, Patient Satisfaction, and Health Care Provider Satisfaction

Patient health, patient satisfaction, and health care provider satisfaction were assessed using structured questionnaires, including patient-reported outcome measures (PROMs) and patient-reported experience measures (PREMs). These questionnaires were emailed to patients and health care providers at predefined time points during the transition process (Table S1 in Multimedia Appendix 1; Table S2 in Multimedia Appendix 2; Multimedia Appendix 3).

PROM surveys were used to assess health-related quality of life. Patient health was assessed with the EQ-5D-5L (EuroQol Group), a generic tool for evaluating health-related quality of life [27]. It consists of 5 items: mobility, self-care, daily activities, pain or discomfort, and anxiety or depression, each rated on a 5-point Likert scale (1=no problems, 5=extreme problems). In addition, overall health was assessed on a scale ranging from 0 (worst health) to 100 (best health), which was normalized to the 5-point scale for consistent analysis. Lower composite scores indicated better patient-reported health.

Patient and health care provider satisfaction was assessed as composite scores based on 5 items: safety, convenience, trust, empowerment, and overall satisfaction. Each item was evaluated through one or more questions, with responses rated on a 7-point Likert scale (1=lowest, 7=highest acceptance). Higher scores reflected greater acceptance of the intervention. As there was no control group, PREMs also included relative questions to capture hypothetical comparative perspectives. Full details of items, response scales, and corresponding questions are provided in Table S1 in Multimedia Appendix 1 and Table S2 in Multimedia Appendix 2.

### Additional Metrics

Discharge rates, readmission rates, and reasons for readmission were obtained from hospital records and the telemedicine provider’s information system.

### Study Outcomes

The primary outcome was patient health and satisfaction during the transition from hospital discharge to home and the first primary care appointment under the structured telephone-based follow-up program. The secondary outcome was health care provider satisfaction, including hospital physicians, TP, and GP. The primary end points were based on the hypothesis that patient health and satisfaction would be considered favorable if they remained stable or improved during the transition process. The secondary end point was based on the hypothesis that health care provider satisfaction would be considered favorable if it remains stable or improves during the transition process, and on whether satisfaction levels differed across health care provider groups.

### Statistical Analysis

Descriptive statistics were used to summarize patient characteristics, reported as frequencies for categorical variables and mean (SD) for continuous variables. Normality of continuous variables was assessed using Q-Q plots. For PROM and PREM scores, descriptive statistics included the mean (SD). If multiple questions formed a single item, the mean item score was calculated and used in the analysis.

Analytical statistics were applied as follows:

- Within-patient changes over time: the paired *t* test were used to assess changes in PROM and PREM scores across time points.
- Between-group comparisons: unpaired *t* tests were used to compare PREM scores across hospital physicians, TPs, and GPs.

The threshold for statistical significance was set at *P*=.05. Effect sizes were reported with 95% CIs. All statistical analyses were performed in RStudio (version 2024.12.1; Posit Software, PBC).

### Ethical Considerations

This study is registered at ClinicalTrials.gov (NCT05617560), which is also listed in the World Health Organization (WHO) International Clinical Trials Registry Platform and the Swiss National Clinical Trial Portal. Ethical approval was obtained by the Ethics Committee Northwest and Central Switzerland (BASEC 2022‐01440). The study was conducted in accordance with the Declaration of Helsinki, the European Medical Device Regulation (EU 2017/745), ISO (International Organization for Standardization) 14155, ISO 14971, ICH-GCP, as well as the Swiss Human Research Act. Prior to enrollment, all participants received information about the study procedures and provided written informed consent. Participants could withdraw at any time without consequence. All personal identifiers were removed prior to data analysis, and all data were handled in accordance with applicable privacy and confidentiality regulations. There was no financial reward or compensation given to the participants.

## Results

### Baseline Characteristics

Between September 2022 and December 2024, we screened a total of 9162 patients at the USB. Of these, 4225 (46.1%) were female and 4918 (53.7%) were male; sex data were missing for 19 (0.2%). Most screenings occurred on internal medicine wards (7290/9162, 79.6%), followed by the emergency unit (1714/9162, 18.7%); 158 cases (1.7%) lacked ward assignment. The mean age was 68.3 (SD 17.4) years; age data were missing in 26 (0.3%) cases. Of all screened patients, 8907 out of 9162 (97.2%) were deemed ineligible, most frequently due to planned physical follow-up (1405/8907, 15.8%), planned rehabilitation (1381/8907, 15.5%), language barriers (1105/8907, 12.4%), or mental disorders (941/8907, 10.6%).

We enrolled a total of 255 out of 9162 (2.8%) patients, of whom 111 out of 255 (43.5%) were female and 144 out of 255 (56.5%) were male. Most were recruited from internal medicine wards (201/255, 78.8%) and the remainder from the emergency unit (54/255, 21.2%). The mean age was 65 (SD 17.6) years. After enrollment, 21 out of 255 (8.2%) patients were excluded prior to first telemedical contact at all due to newly identified exclusion criteria and classified as screening failures.

**Figure 2.**
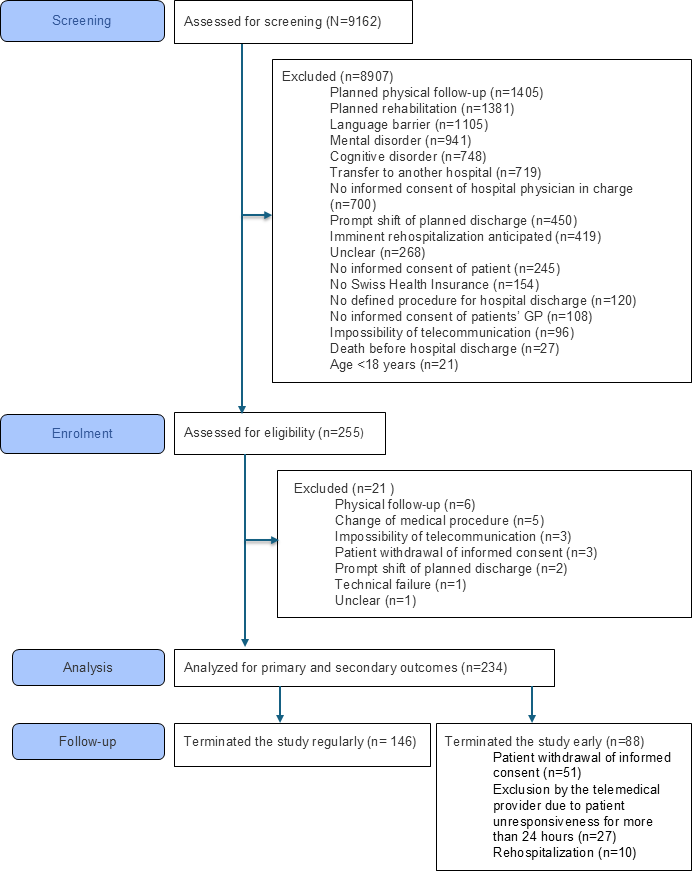
CONSORT (Consolidated Standards of Reporting Trials) flow diagram illustrating the screening and recruitment procedure, including the absolute number of participants at each stage (n) and reasons for exclusion expressed in absolute numbers (n). GP: general practitioner.

Consequently, we included 234 patients in the final analysis (101/234, 43.2% female; 133/234, 56.8% male); mean age was 64.8 (SD 17.5) years. Among these patients, 25 out of 234 (10.7%) reported not having a GP, 70 out of 234 (29.9%) lived alone, and 162 out of 234 (69.2%) either with a partner, family, or friends; data were missing for 2 out of 234 (0.9%) patients. Per-protocol completion was achieved by 146 out of 234 (62.4%) patients. A total of 88 out of 234 (37.6%) patients discontinued early due to withdrawal of consent (51/234, 21.8%), telephone unavailability for >24 (27/234, 11.5%) hours, or rehospitalization (10/234, 4.3%; Figure 2; Table 1).

**Table 1.** Baseline characteristics of all participants (N=234), including completers and early dropouts.

Characteristics	Regular termination	Early termination			Total
Patient incompliance	Patient withdrawal	Rehospitalization
Participants, N	146	27	51	10	234
Age (years), mean (SD)	63.9 (17)	61.6 (18.9)	70.5 (16.2)	56.5 (22)	64.8 (17.5)
Sex, n (%)				
Female	69 (47.3)	11 (40.7)	16 (31.4)	5 (50)	101 (43.2)
Male	77 (52.7)	16 (59.3)	35 (68.6)	5 (50)	133 (56.8)
Hospital stay, n (%)				
Emergency unit	27 (18.5)	8 (29.6)	11 (21.6)	3 (30.0)	49 (20.9)
Internal medicine ward	119 (81.5)	19 (70.4)	40 (78.4)	7 (70.0)	185 (79.1)
General practitioner, n (%)				
Yes	132 (90.4)	21 (77.8)	46 (90.2)	9 (90.0)	208 (88.9)
No	13 (8.9)	6 (22.2)	5 (9.8)	1 (10.0)	25 (10.7)
Missing data	1 (0.7)	—^a^	—	—	1 (0.4)
Primary diagnosis, n (%)				
Allergological	1 (0.7)	0 (0)	0 (0)	0 (0)	1 (0.4)
Angiological	1 (0.7)	0 (0)	0 (0)	0 (0)	1 (0.4)
Dermatological	1 (0.7)	0 (0)	0 (0)	0 (0)	1 (0.4)
Endocrinological	2 (1.4)	1 (3.7)	1 (2.0)	1 (10.0)	5 (2.1)
Gastroenterological	12 (8.2)	1 (3.7)	2 (4.0)	0 (0)	15 (6.4)
Hematological	0 (0)	0 (0)	2 (4.0)	0 (0)	2 (0.9)
ENT^b^	3 (2.1)	0 (0)	1 (2.0)	0 (0)	4 (1.7)
Infectious	14 (9.6)	3 (11.1)	9 (17.6)	1 (10.0)	27 (11.5)
Cardiological	36 (24.7)	8 (29.6)	10 (19.6)	2 (20.0)	56 (23.9)
Nephrological	1 (0.7)	0 (0)	0 (0)	1 (10.0)	2 (0.9)
Neurological	11 (7.5)	1 (3.7)	5 (9.8)	0 (0)	17 (7.3)
Pulmonary	29 (19.9)	3 (11.1)	6 (11.8)	3 (30.0)	41 (17.5)
Rheumatological	4 (2.7)	0 (0)	0 (0)	0 (0)	4 (1.7)
Accident	10 (6.8)	1 (3.7)	5 (9.8)	0 (0)	16 (6.8)
Urological	2 (1.4)	0 (0)	3 (5.9)	1 (10.0)	6 (2.6)
No confirmed primary diagnosis	19 (13.0)	9 (33.3)	7 (13.7)	1 (10.0)	36 (15.4)
Living situation, n (%)				
Alone	42 (28.8)	8 (29.6)	19 (37.3)	1 (10.0)	70 (29.9)
Family members or friends	102 (69.9)	19 (70.4)	32 (62.7)	9 (90.0)	162 (69.2)
Missing data	2 (1.4)	—	—	—	2 (1.0)
Family situation, n (%)				
Divorced	11 (7.5)	3 (11.1)	3 (5.9)	1 (10.0)	18 (7.7)
Married	84 (57.5)	15 (55.6)	30 (58.8)	7 (70.0)	136 (58.1)
Separated	6 (4.1)	0 (0)	1 (2.0)	0 (0)	7 (3.0)
Single	30 (20.5)	6 (22.2)	14 (27.5)	2 (20.0)	52 (22.2)
Widowed	14 (9.6)	2 (7.4)	3 (5.9)	0 (0)	19 (8.1)
Missing data	1 (0.7)	1 (3.7)	—	—	2 (0.9)
SPI^c^ and ePA-AC^d^^,^^e^				
Count, n (%)	117/146 (80.1)	19/27 (70.4)	40/51 (78.4)	7/10 (70.0)	183/234 (78.2)
SPI score, mean (SD)	38.6 (3.1)	39.1 (1.8)	38.7 (2.4)	38.6 (2.1)	38.7 (2.8)
ePA-AC, mean (SD)				
Upper body undressing and dressing	3.9 (0.4)	3.9 (0.2)	3.9 (0.3)	3.7 (0.5)	3.9 (0.4)
Lower body undressing/and dressing	3.8 (0.5)	3.9 (0.2)	3.8 (0.5)	3.7 (0.5)	3.8 (0.5)
Locomotion	3.8 (0.5)	3.8 (0.4)	3.7 (0.6)	3.9 (0.4)	3.8 (0.5)
Upper body care	3.9 (0.4)	3.9 (0.2)	3.8 (0.4)	3.7 (0.5)	3.9 (0.4)
Lower body care	3.7 (0.6)	3.8 (0.4)	3.7 (0.6)	3.7 (0.5)	3.7 (0.6)
Eating	4 (0.2)	3.9 (0.2)	4 (0.2)	3.9 (0.4)	4 (0.2)
Drinking	4 (0.3)	4 (0)	4 (0.2)	4 (0)	4 (0.3)
Stool excretion	3.9 (0.3)	3.8 (0.7)	4 (0.2)	4 (0)	3.9 (0.4)
Urine excretion	3.9 (0.4)	3.8 (0.7)	4 (0.2)	4 (0)	3.9 (0.4)
Change in body position	3.9 (0.4)	3.9 (0.2)	4 (0.2)	4 (0)	3.9 (0.3)

a Not available.

b ENT: ear, nose, and throat.

c SPI: Self-Care Performance Index.

d ePA-AC: the outcome-based nursing assessment for acute care.

e SPI score (0-40) from the ePA‑AC (10 questions, 0-4) assesses care needs, with lower scores indicating greater dependency.

### Patient-Reported Health Outcomes

**Figure 3.**
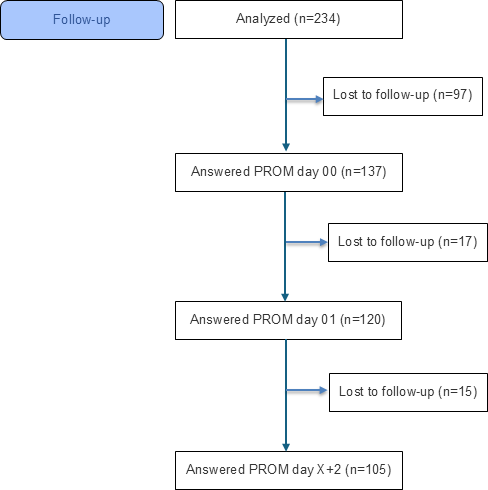
CONSORT (Consolidated Standards of Reporting Trials) flow diagram illustrating the patient-reported outcome measure follow-up, including the absolute number of participants at each stage who completed the questionnaires and those who were lost to follow-up (n). PROM: patient-reported outcome measure.

Response rates for PROMs were 137 out of 234 (58.5%) at baseline (day 00), 120 out of 234 (51.3%) after the first teleconsultation (day 01), and 105 out of 234 (44.9%) after the last teleconsultation (day X+2; Figure 3).

### Changes in Composite Scores

**Figure 4.**
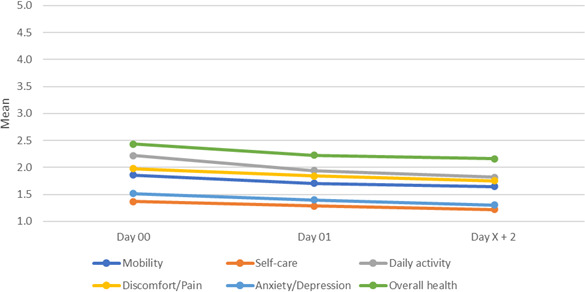
Line chart showing mean patient-reported health scores over time across 6 items.

Composite health scores (mobility, self-care, daily activities, pain or discomfort, anxiety or depression, and overall health; Likert 1‐5) improved significantly from baseline to final follow-up (mean difference –0.227, SD 0.334; *t*_93_=−6.60; *P*<.001; 95% CI −0.296 to −0.159), from day 00 to day 01 (mean difference −0.137, SD 0.375; *t*_104_=–3.75; *P*<.001; 95% CI −0.210 to -0.064) and from day 01 to day X+2 (mean difference −0.101, SD 0.357; *t*_94_=–2.77; *P*=.007; 95% CI −0.174 to -0.029). Patient-reported health did not deteriorate at any time (Table 2; Figure 4).

**Table 2.** Patient-reported health outcomes^a^, including 6 items and the composite, are presented as mean (SD).

Items	Day 00, mean (SD)	Day 01, mean (SD)	Day X + 2, mean (SD)
Mobility	1.9 (0.9)	1.7 (0.8)	1.6 (0.8)
Self-care	1.4 (0.8)	1.3 (0.7)	1.2 (0.6)
Daily activity	2.2 (1.1)	1.9 (1)	1.8 (0.9)
Discomfort or pain	2.0 (1)	1.8 (0.9)	1.8 (0.8)
Anxiety or depression	1.5 (0.8)	1.4 (0.7)	1.3 (0.7)
Overall health^b^	2.4 (0.8)	2.2 (0.8)	2.2 (0.8)
Total	1.9 (0.7)	1.7 (0.6)	1.6 (0.6)

a Patient health outcomes were assessed using patient-reported outcome measure questionnaires with a 5-point Likert scale (1=best, 5=worst) at day 00, day 01, and day X + 2.

b Overall health scores, originally normed on a 0-100 scale, were rescaled to a 5-point scale for comparability.

### Patient-Reported Satisfaction Outcomes

**Figure 5.**
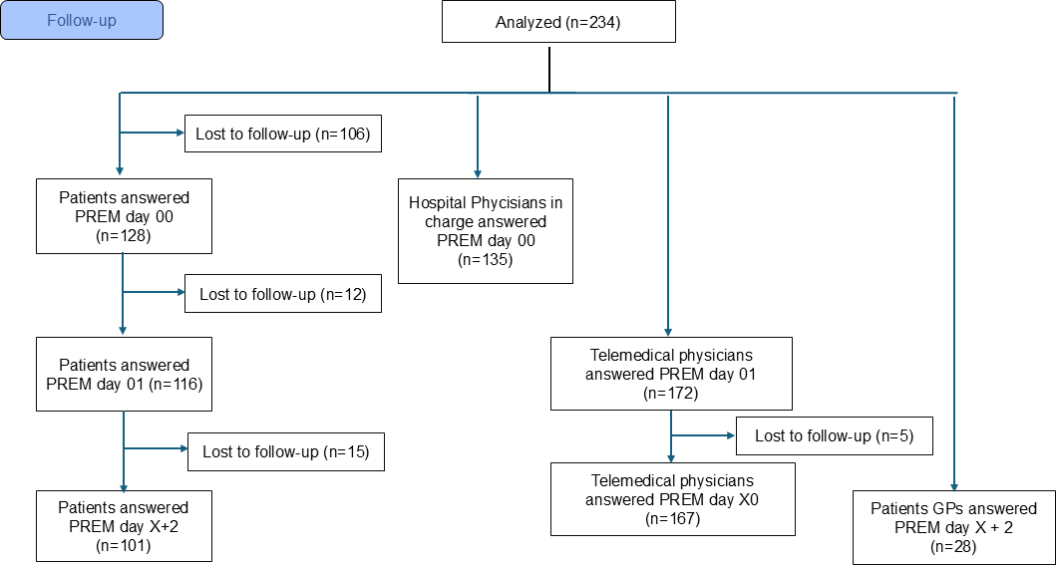
CONSORT (Consolidated Standards of Reporting Trials) flow diagram illustrating the patient-reported experience measure follow-up, including the absolute number of participants at each stage who completed the questionnaires and those who were lost to follow-up (n). GP: general practitioner; PREM: patient-reported experience measure.

PREM response rates were 128 out of 234 (54.7%) at day 00, 116 out of 234 (49.6%) at day 01, and 101 out of 234 (43.2%) at day X+2 (Figure 5).

### Changes in Composite Scores

**Figure 6.**
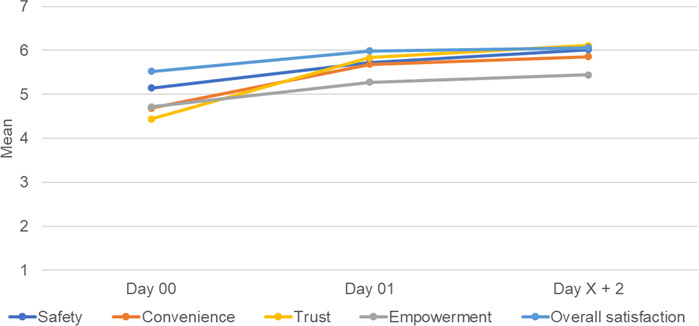
Line chart showing mean patient-reported satisfaction scores over time across 5 items.

Composite satisfaction scores (safety, convenience, trust, empowerment, and overall satisfaction; Likert 1‐7) improved significantly between day 00 and day 01 (mean difference 0.443. SD 1.058; *t*_95_=4.10; *P*<.001; 95% CI 0.228‐0.657), from day 00 to day X+2 (mean difference 0.714. SD 1.031; *t*_87_=6.50; *P*<.001; 95% CI 0.496‐0.933) and between day 01 and day X+2 (mean difference 0.176, SD 0.606; *t*_92_=2.80; *P*=.006; 95% CI 0.051-0.300). Patient-reported satisfaction did not deteriorate at any time (Table 3; Figure 6).

**Table 3.** Patient- and stakeholder-reported satisfaction^a^ outcomes, including 5 items and the composite, are presented as mean (SD).

Items	Day 00. mean (SD)		Day 01, mean (SD)		Day X0, mean (SD)	Day X + 2, mean (SD)	
	Patient	Hospital	Patient	TP^b^	TP	Patient	GP^c^
Safety	5.2 (1.6)	5.7 (1.1)	5.7 (1.2)	5.8 (1.6)	5.7 (1.7)	6 (1.2)	4.6 (2.1)
Convenience	4.7 (1.3)	5.5 (1)	5.7 (1)	5.8 (1.2)	5.8 (1.3)	5.9 (0.9)	3.8 (1.6)
Trust	4.5 (2.1)	3.9 (1.8)	5.8 (1.1)	5.9 (1.5)	5.7 (1.7)	6.1 (1.1)	3.5 (1.8)
Empowerment	4.8 (1.8)	4.8 (1.4)	5.3 (1.8)	6.1 (1.2)	6 (1.4)	5.4 (1.8)	3.8 (1.8)
Overall satisfaction	5.6 (1.5)	5.5 (1.1)	6 (1.3)	6.3 (1)	6.2 (1)	6.1 (1.3)	3.8 (1.7)
Total	5 (1.4)	5.1 (1)	5.7 (1.1)	6 (1)	5.9 (1.1)	5.9 (1.1)	3.9 (1.6)

a Patient and health care provider satisfaction outcomes were assessed using patient-reported experience measure questionnaires with 7-point Likert scale (1=lowest, 7=highest).

b TP: telemedical physician.

c GP: general practitioner.

### Detailed Insights

We classified patient-rated satisfaction as positive if scored ≥5. At baseline, 69.5% (89/128) reported feeling medically safe, increasing to 85.3% (99/116) after the first consultation and 90.1% (91/101) at follow-up. Similarly, the transfer process was rated as easy by 71.9% (92/128) at baseline, 86.2% (100/116) at day 01, and 87.1% (88/101) at follow-up. Telemedical monitoring was perceived as facilitating the transition by 86.2% (100/116) at day 01 and 85.1% (86/101) at follow-up, while feeling well looked after was reported by 85.3% (99/116) and 90.1% (91/101), respectively. Perceived independence in daily life improved from 59.4% (76/128) at baseline to 75.9% (88/116) at day 01 and 78.2% (79/101) at follow-up. Overall satisfaction followed a similar pattern, increasing from 76.6% (98/128) at baseline to 88.8% (103/116) at day 01 and 91.1% (92/101) at follow-up (Table S1 in Multimedia Appendix 1).

### Health Care Provider–Reported Satisfaction Outcomes

#### Response Rates

Health care providers (hospital physicians, TPs, and GPs) returned 135 out of 234 (57.7%), 172 out of 234 (73.5%), 167 out of 234 (71.4%), and 28 out of 234 (13.5%) questionnaires at predefined time points (Figure 5).

#### Between-Group Changes in Composite Scores

**Figure 7.**
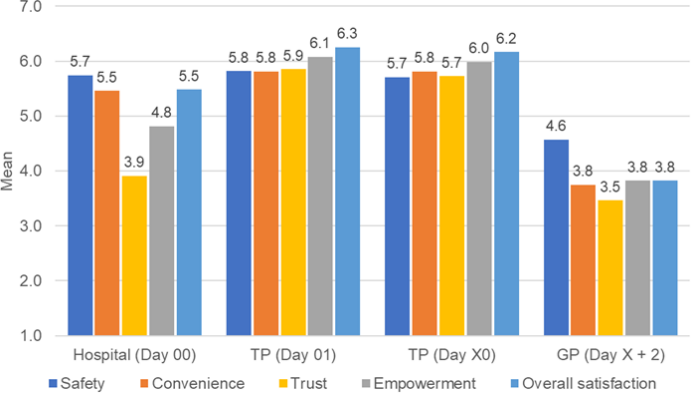
Column chart showing mean health care provider–reported satisfaction scores across 5 items.

Composite satisfaction scores differed significantly between groups: higher for hospital physicians (mean 5.081, SD 0.978) than GPs (mean 3.886, SD 1.613; *t*_31.24_=3.78; *P*<.001; 95% CI 0.551‐1.841), higher for TP (mean 5.919, SD 0.831) than hospital physicians (mean 5.081, SD 0.978; *t*_262.8_=7.95; *P*<.001; 95% CI 0.630‐1.045), and higher for TPs (mean 5.919, SD 0.831) than GPs (mean 3.886, SD 1.613; *t*_29.38_=6.53; *P*<.001; 95% CI 1.397‐2.667; Table 3; Figure 7).

### GP Satisfaction: Detailed Insights

We classified ratings as negative if scored ≤3. Among 28 GPs, 7 (25%) rated the transfer process as difficult, and 9 (32.1%) reported incomplete patient information at the required time. More than half (15/28, 53.6%) perceived less to no added value for themselves or their patients compared with usual discharge. Nearly half (13/28, 46.4%) reported insufficient communication and were unlikely to recommend telemedical support. Perceived patient independence and overall satisfaction were low (10/28, 35.7%; Table S2 in Multimedia Appendix 2).

## Discussion

### Principal Findings

We implemented and evaluated a telephone-based follow-up program to support patient health and satisfaction as well as health care provider satisfaction during the transition from hospital discharge to home and the first primary care appointment.

Of 9162 screened patients, 255 (2.8%) were enrolled, and 234 (2.6%) were included in the final analysis. Per-protocol completion was achieved by 146 out of 234 (62.4%) patients, whereas 88 out of 234 (37.6%) patients discontinued early due to withdrawal of consent, telephone unavailability for >24 hours, or rehospitalization (Table 1; Figure 2). Patient-reported health and satisfaction changed during follow-up, while no deterioration was observed (Tables2  3; Figures4  6). Health care provider satisfaction differed across groups, with TPs reporting the highest and GPs the lowest levels, citing challenges in information transfer and perceived added value (Table 3; Figure 7).

### Comparison With Previous Work: Differences From Standard Follow-Up Care

In standard care, patients typically attend an in-person primary care follow-up after discharge, with waiting times depending on GP workload but ideally within 10 days, as reported by Riverin et al [28]. In contrast, participants in our study were proactively contacted by telephone to schedule their first teleconsultation within 24 hours of discharge. This approach standardized the transition, eliminating the need for patients to arrange care or travel. Additionally, patients had 24/7 access to the telemedical provider for immediate medical support.

The telemedical follow-up program was not designed to accelerate hospital discharge but to bridge the vulnerable postdischarge period until the first GP visit [1-4,8]. It aimed to reduce the workload of GPs and their covering physicians by remotely managing suitable patients and helping to prevent unplanned primary care visits, as follow-up needs after discharge are often urgent. Hospital physicians largely supported this intention, although their PREM ratings also reflected some indecision about the value of telemedicine for discharge planning. Conversely, GPs reported lower perceived benefit for practice management (Table S2 in Multimedia Appendix 2), consistent with the lower composite satisfaction scores among GPs (Table 3; Figure 7). However, given the small GP sample (28 participants), these differences may be unstable and should be interpreted with caution. Although cost analysis was not the primary objective of this study, the program incurred higher expenditures due to the complex recruitment procedures inherent to clinical trial settings. Nevertheless, it is anticipated to be cost-neutral—or potentially cost-saving—under routine care conditions by alleviating GPs’ workload without increasing consultation frequency. Given the limited evidence on telemedicine facilitating transitions from hospital to primary care, further research is warranted to comprehensively evaluate its economic implications.

### Feasibility of the Intervention

Feasibility was constrained by a low inclusion rate of 2.8%, largely due to the study design. All patients hospitalized in the emergency unit and internal medicine wards were screened, with many excluded because of strict eligibility criteria. Among these, nearly half (4374/8907, 49.2%) required some form of physical treatment, including planned physical follow-up, planned rehabilitation, transfer to another hospital, prompt changes in discharge plans, or anticipated imminent rehospitalization (Figure 1). The acute care profile of the USB, which treats a disproportionately high number of patients with complex medical conditions, contributed indirectly to the low inclusion rate, as many of these patients could not be discharged directly home.

Recruitment was further hindered by procedural barriers. The responsible hospital physician first had to confirm eligibility, followed by obtaining consent from the patient’s GP. Delays in these steps occasionally rendered patients ineligible due to changes in their medical plans. Finally, informed consent had to be obtained from the patient.

Despite these challenges, 62.4% of included patients adhered to the program per protocol, demonstrating feasibility for selected patient groups. Based on our findings, the intervention appears most suitable for patients who (1) are aged 18 years or older; (2) have cardiological, pulmonary, or infectious diseases; (3) are able to communicate via telephone and email; (4) possess adequate language proficiency and cognitive or mental capacity; and (5) have a discharge plan appropriate for remote follow-up, including no planned physician follow-up appointments, no planned rehabilitation stays, no transfers to another hospital, no imminent rehospitalization is anticipated, and no need for tests that must be conducted in a health care institution (laboratory testing, ECG, etc).

### Early Discontinuation: Nonresponsiveness

Failure to establish contact within 24 hours after discharge led to exclusion per protocol. During follow-up, 27/234 (11.5%) patients could not be reached (Figure 2). This represented an uncomfortable situation for TPs who were unable to determine the underlying cause. Technical or personal factors were most implicated, as identified through proactive outreach by the study team; however, medical emergencies cannot be ruled out.

This finding highlights the potential added value of integrating wearable sensors (eg, vital signs) in the early postdischarge period. They provide complementary information beyond the importance of physiological data to patients and health care providers. Future research should investigate the determinants of patient unreachability and, more broadly, the reasons for withdrawal. This could help improve telemedical follow-up program design and enhance patient acceptance, as also emphasized by Bjarnadóttir et al [24].

### Early Discontinuation: Rehospitalization

Rehospitalization occurred in 10 out of 234 (4.3%) patients in this study, higher than the rate observed among contemporaneously discharged patients from the USB without telemedical follow-up (2.8%). While this finding may suggest potential limitations of the intervention, it may also reflect improved early detection of clinical worsening due to 24/7 accessibility to telemedicine. Rehospitalizations in this study were often initiated to rule out acute medical emergencies following the onset or worsening of symptoms, ensuring patient safety. This aligns with previous research demonstrating a higher demand for acute health care services among patients with acute conditions receiving telemedical follow-up. In contrast, readmission rates tend to be lower among patients with chronic diseases [29-31]. Notably, 17.5% (41/234) of patients in this study were diagnosed with pulmonary diseases (Table 1), and respiratory symptoms were the main cause of rehospitalization in 40% of cases, mirroring trends observed in prior studies [31].

In contrast, inherent limitations of remote care, such as reduced diagnostic capability as well as communication and technical challenges, may also have led to diagnostic uncertainty and influenced rehospitalization decisions. These factors are consistent with findings from Shah et al [32], who reported similar challenges in their evaluation of telehealth interventions.

Furthermore, unlike the rehospitalization rate reported by the USB, which includes only readmissions to its own facility within 18 days after hospital discharge, our study also captured rehospitalizations to external hospitals. This broader inclusion criterion may have contributed to the higher observed rate.

### Impact on Patient Health and Satisfaction

Changes were observed in patient-reported health and satisfaction during the follow-up period, with no deterioration noted (Tables2  3; Figures4  6). These findings are consistent with previous reports of high patient satisfaction with telehealth [33,34].

However, the observed changes may also have been influenced by contextual factors beyond the telemedicine-based intervention itself (Table 1). Natural recovery after discharge, coupled with the fact that most participants were married or lived with family members who could support their self-care, potentially contributed to higher patient-reported health scores. Additionally, the mean SPI score was 38.7 (SD 2.8), indicating relatively high baseline functional status (Table 1). Nevertheless, evidence from recent studies suggests that patients with lower functional capacity may also benefit from telemedical follow-up [35].

### Impact on Health Care Provider Satisfaction

Health care provider satisfaction differed across groups (Table 3; Figure 7). Although GP consent was obtained to support collaboration, they reported lower satisfaction levels, which may reflect limited familiarity with telemedicine or concerns regarding coordination and responsibility. In contrast, TPs who were more experienced with telemedicine workflows reported higher satisfaction levels. It should be noted that the GP sample was small (28 participants), and the observed differences may be unstable. These results should therefore be interpreted with caution. Overall, the findings indicate variability in provider experiences and highlight the need for further research to better understand factors influencing satisfaction across professional groups. Strengthening engagement, improving interoperability between hospital, telemedical, and primary care services, and addressing provider-specific concerns may help improve the acceptability and sustainability of telemedical follow-up programs.

### Clinical and Digital Implications

This study on a telephone-based follow-up program demonstrates how structured postdischarge support can inform digital health policy and hospital discharge workflows. The program may serve as a model for a unified hospital follow-up system and standardized post discharge care management. It enables targeted resource use in hospitals and primary care by reducing unplanned visits and saving clinician time through structured, standardized processes. The intervention has the potential to reduce costs and improve patient safety during the vulnerable postdischarge period, while continuous support facilitates the transition to home and enhances patient recovery. Moreover, feasibility results provide a foundation for more complex digital interventions (eg, continuous vital sign monitoring) by identifying both benefits and challenges. Finally, findings on patient and health care provider satisfaction provide important insights for digital health policy, supporting the development of follow-up programs that meet the needs of all stakeholders. As noted by Chen et al [36], it is important to continually revise such models, as technology evolves rapidly, shaping individuals’ expectations of the possibilities offered by telemedicine.

### Limitations

This study has several limitations. First, the study was conducted at a single center without a comparison to usual care, which may limit the generalizability of the findings to other clinical settings and constrain conclusions about the intervention’s effectiveness. Second, participants were recruited exclusively from the internal medicine wards and the emergency unit, which may restrict the applicability of the results to other patient populations. Third, despite a per-protocol completion rate of more than half, the low inclusion rate—driven by multiple exclusion criteria—and the very high functional status of enrolled patients resulted in a highly selective sample. Consequently, the intervention’s generalizability and feasibility are limited, as it could not be delivered to the majority of discharged patients. Fourth, the technical infrastructure required for routine care is not yet fully established, which may affect the feasibility of integrating this telemedicine-based intervention into everyday clinical practice at present. Fifth, although the categories for early discontinuation were documented, a detailed exploration of the underlying causes was beyond the scope of this study. Sixth, given natural recovery after discharge and other concurrent care, improvements in PROMs and PREMs cannot be solely attributed to the telemedical program. Finally, the frequency distribution of scores in PROMs and PREMs should be interpreted with caution, as the loss to follow-up may have introduced response bias that could lead to an overestimation of favorable outcomes. Patients who were more favorable toward the study were likely to remain enrolled and complete the questionnaires, whereas those less engaged were more likely to discontinue participation and not complete them.

### Conclusion

This study outlines both the potential benefits and the practical challenges of implementing a telephone follow-up program to support the transition from hospital to home and primary care. Variations in physician satisfaction underscore the need for a more user-friendly technical infrastructure and clearer role definitions across providers. Future research should be conducted in multicenter settings, involving a broader patient population and a control group receiving usual care. Moreover, efforts should be directed toward streamlining the recruitment process to enable the inclusion of a larger study population, thereby improving the feasibility and generalizability of the findings.

## Supplementary material

10.2196/85467 Multimedia Appendix 1 Patient-reported satisfaction ratings (n, %) across 5 items (day 00: n=128, day 01: n=116, day X + 2: n=101).

10.2196/85467 Multimedia Appendix 2 Health care provider-reported satisfaction ratings (n, %) for each question across 5 items (hospital physician: n=135, TP day 01: n=172, TP day X0: n=167, GP: n=28).

10.2196/85467 Multimedia Appendix 3 Questionnaires.

10.2196/85467 Checklist 1 CONSORT checklist.

## Abbreviations

ePA-AC: outcome-based nursing assessment for acute care
GP: general practitioner
ISO: International Organization for Standardization
PREM: patient-reported experience measure
PROM: patient-reported outcome measure
SHIFT: Smart Hospital: Integrated Framework, Tools, and Solutions
SPI: Self-Care Performance Index
TP: telemedical physician
USB: University Hospital of Basel
WHO: World Health Organization
