# Supplementary material for: Evaluating a Telemedical Follow-Up Program for Continuity of Care After Hospital Discharge: Prospective Clinical Intervention Study
Source: JMIR Form Res. 2026 Mar 27;10:e85467. doi: 10.2196/85467 (PMC13026428; doi:10.2196/85467)
Supplement: Multimedia Appendix 1 [file formative-v10-e85467-s001.docx]

**Multimedia Appendix 1**

|  | **Safety** | | | | | | | |
| --- | --- | --- | --- | --- | --- | --- | --- | --- |
| **Question^a^** | **Day 00** | **Day 01** | **Day X+2** | **Score, n (%)** | **Day 00** | **Day 01** | | **Day X+2** |
| **Q1** | I feel medically well cared with telemedical support for the transfer to home. | I feel medically safe with the telemedical support. | I felt medically safe with the telemedical support. | **1**  **2**  **3**  **4**  **5**  **6**  **7**  **NA** | 5 (3.9)  6 (4.7)  7 (5.5)  20 (15.6)  25 (19.5)  32 (25)  32 (25)  1 (0.8) | 1 (0.9)  1 (0.9)  2 (1.7)  13 (11.2)  27 (23.3)  36 (31)  36 (31)  0 (0) | 1 (1)  0 (0)  2 (2)  7 (6.9)  18 (17.8)  28 (27.7)  45 (44.6)  0 (0) | |
|  | **Convenience** | | | | | | | |
| **Question** | **Day 00** | **Day 01** | **Day X+2** | **Score, n (%)** | **Day 00** | **Day 01** | **Day X+2** | |
| **Q1** | I find the transfer process from hospital to telemedical care easy. | I find the transfer process from hospital to telemedical care easy. | I found the transfer process from hospital to telemedical support easy. | **1**  **2**  **3**  **4**  **5**  **6**  **7**  **NA** | 3 (2.3)  6 (4.7)  10 (7.8)  16 (12.5)  24 (18.8)  33 (25.8)  35 (27.3)  1 (0.8) | 2 (1.7)  4 (3.5)  7 (6)  3 (2.6)  18 (15.5)  36 (31)  46 (39.7)  0 (0) | 2 (2)  1 (1)  5 (5)  5 (5)  8 (7.9)  36 (35.6)  44 (43.6)  0 (0) | |
| **Q2** | Compared to what I have experienced with others when I was transferred home, telemedical support adds value for me. | I found the information regarding my treatment from all medical professionals involved (hospital, telemedical support, primary care physician) to be consistent. | I found the information regarding my treatment from all medical professionals involved (hospital, telemedical support, primary care physician) to be consistent. | **1**  **2**  **3**  **4**  **5**  **6**  **7**  **NA** | 2 (1.6)  0 (0)  0 (0)  4 (3.1)  6 (4.7)  2 (1.6)  3 (2.3)  111 (86.7) | 2 (1.7)  0 (0)  3 (2.6)  10 (8.6)  21 (18.1)  36 (31)  44 (37.9)  0 (0) | 1 (1)  1 (1)  4 (4)  12 (11.9)  11 (10.9)  38 (37.6)  34 (33.7)  0 (0) | |
| **Q3** | Compared to being transferred home from previous hospital stays, telemedical monitoring adds value for me. | Telemedical monitoring made the transition from hospital to home easier for me. | Telemedical monitoring made the transition from hospital to home easier for me. | **1**  **2**  **3**  **4**  **5**  **6**  **7**  **NA** | 10 (7.8)  8 (6.3)  7 (5.5)  23 (18)  25 (19.5)  17 (13.3)  20 (15.6)  18 (14.1) | 3 (2.6)  2 (1.7)  5 (4.3)  6 (5.2)  16 (13.8)  41 (35.3)  43 (37.1)  0 (0) | 2 (2)  4 (4)  2 (2)  7 (6.9)  16 (15.8)  33 (32.7)  37 (36.6)  0 (0) | |
| **Q4** | I slept well during the inpatient stay. | I slept well at home last night. | I slept well at home the last few nights. | **1**  **2**  **3**  **4**  **5**  **6**  **7**  **NA** | 6 (4.7)  21 (16.4)  23 (18)  20 (15.6)  25 (19.5)  22 (17.2)  11 (8.6)  0 (0) | 3 (2.6)  2 (1.7)  5 (4.3)  6 (5.2)  16 (13.8)  41 (35.3)  43 (37.1)  0 (0) | 1 (1)  0 (0)  5 (5)  5 (5)  13 (12.9)  41 (40.6)  36 (35.6)  0 (0) | |
|  | **Trust** | | | | | | | |
| **Question/Timepoint** | **Day 00** | **Day 01** | **Day X+2** | **Score, n (%)** | **Day 00** | **Day 01** | **Day X+2** | |
| **Q1** | I know what the next steps in the telemedical support are. | I know what the next steps in the telemedical support are. |  | **1**  **2**  **3**  **4**  **5**  **6**  **7**  **NA** | 19 (14.8)  8 (6.3)  10 (7.8)  16 (12.5)  19 (14.8)  27 (21.1)  26 (20.3)  3 (2.3) | 7 (6)  5 (4.3)  5 (4.3)  10 (8.6)  19 (16.4)  23 (19.8)  47 (40.5)  0 (0) |  | |
| **Q2** |  | I feel well looked after by the telemedical support. | I felt well looked after by the telemedical support. | **1**  **2**  **3**  **4**  **5**  **6**  **7**  **NA** |  | 2 (1.7)  1 (0.9)  2 (1.7)  12 (10.3)  14 (12.1)  36 (31)  49 (42.2)  0 (0) | 1 (1)  0 (0)  2 (2)  7 (6.9)  12 (11.9)  31 (30.7)  48 (47.5)  0 (0) | |
| **Q3** |  | I know who I can turn to if I have any questions. |  | **1**  **2**  **3**  **4**  **5**  **6**  **7**  **NA** |  | 2 (1.7)  1 (0.9)  4 (3.5)  8 (6.9)  11 (9.5)  22 (19.0)  68 (58.6)  0 (0) |  | |
|  | **Empowerment** | | | | | | | |
| **Question** | **Day 00** | **Day 01** | **Day X+2** | **Score, n (%)** | **Day 00** | **Day 01** | **Day X+2** | |
| **Q1** | With the help of telemedical support, I will be able to shape my everyday life independently | With the help of telemedical support, I can shape my everyday life independently. | With the help of telemedical support, I was able to shape my everyday life independently. | **1**  **2**  **3**  **4**  **5**  **6**  **7**  **NA** | 10 (7.8)  9 (7)  5 (3.9)  26 (20.3)  22 (17.2)  31 (24.2)  23 (18)  2 (1.6) | 8 (6.9)  5 (4.3)  6 (5.2)  9 (7.8)  25 (21.6)  26 (22.4)  37 (31.9)  0 (0) | 7 (6.9)  3 (3)  2 (2)  10 (9.9)  21 (20.8)  20 (19.8)  38 (37.6)  0 (0) | |
|  | **Overall Satisfaction** | | | | | | | |
| **Question/Timepoint** | **Day 00** | **Day 01** | **Day X+2** | **Score, n (%)** | **Day 00** | **Day 01** | **Day X+2** | |
| **Q1** | I think that telemedical support is a good offer. | I am satisfied with the telemedical support offered. | I am satisfied with the telemedical support offered. | **1**  **2**  **3**  **4**  **5**  **6**  **7**  **NA** | 2 (1.6)  6 (4.7)  2 (1.6)  18 (14.1)  20 (15.6)  31 (24.2)  47 (36.7)  2 (1.6) | 2 (1.7)  1 (0.9)  1 (0.9)  9 (7.8)  20 (17.2)  29 (25.0)  54 (46.6)  0 (0) | 2 (2)  1 (1)  1 (1)  5 (5)  15 (14.9)  29 (28.7)  48 (47.5)  0 (0) | |

***^a^*** Questions across all items were rated *on a seven-point Likert scale (1 = lowest, 7 = highest).*
