# Supplementary material for: Evaluating a Telemedical Follow-Up Program for Continuity of Care After Hospital Discharge: Prospective Clinical Intervention Study
Source: JMIR Form Res. 2026 Mar 27;10:e85467. doi: 10.2196/85467 (PMC13026428; doi:10.2196/85467)
Supplement: Multimedia Appendix 2 [file formative-v10-e85467-s002.docx]

**Multimedia Appendix 2**

|  | **Safety** | | | | | | | | |
| --- | --- | --- | --- | --- | --- | --- | --- | --- | --- |
| **Questions^a^** | **Hospital physician** | **TP**  **(Day 01)** | **TP**  **(Day X0)** | **GP** | **Score, n (%)** | **Hospital physician** | **TP**  **(Day 01)** | **TP**  **(Day X0)** | **GP** |
| **Q1** | I feel that this patient is suitable for tele-medical support. | I feel that this patient is suitable for tele-medical support | I feel that this patient was suitable for tele-medical support | I feel that this patient was suitable for tele-medical support | **1**  **2**  **3**  **4**  **5**  **6**  **7**  **NA** | 0 (0)  4 (3)  2 (1.5)  9 (6.7)  31 (23)  53 (39.3)  36 (26.7)  0 (0) | 5 (2.9)  5 (2.9)  6 (3.5)  14 (8.1)  22 (12.8)  38 (22.1)  82 (47.7)  0 (0) | 7 (4.2)  7 (4.2)  7 (4.2)  16 (9.6)  16 (9.6)  31 (18.6)  83 (49.7)  0 (0) | 4 (14.3)  2 (7.1)  2 (7.1)  5 (17.9)  3 (10.7)  5 (17.9)  7 (25)  0 (0) |
|  | **Convenience** | | | | | | | | |
| **Questions** | **Hospital physician** | **TP**  **(Day 01)** | **TP**  **(Day X0)** | **GP** | **Score, n (%)** | **Hospital physician** | **TP**  **(Day 01)** | **TP**  **(Day X0)** | **GP** |
| **Q1** | I find the process of trans-ferring this patient from hospital to tele-medical care easy. | I find the process of trans-ferring this patient from hospital to tele-medical care easy. | I find the process of trans-ferring this patient from telemedical support to the family doctor straightforward | I find the transfer process from tele-medical support to the family doctor easy. | **1**  **2**  **3**  **4**  **5**  **6**  **7**  **NA** | 0 (0)  1 (0.7)  0 (0)  14 (10.4)  34 (25.2)  51 (37.8)  35 (25.9)  0 (0) | 4 (2.3)  4 (2.3)  7 (4.1)  8 (4.7)  30 (17.4)  47 (27.3)  72 (41.9)  0 (0) | 2 (1.2)  2 (1.2)  10 (6)  13 (7.8)  21 (12.6)  36 (21.6)  83 (49.7)  0 (0) | 4 (14.3)  1 (3.6)  2 (7.1)  5 (17.9)  6 (21.4)  7 (25)  3 (10.7)  0 (0) |
| **Q2** |  | All relevant patient in-formation was available to me at the required time. | All relevant patient in-formation was available to me at the required time. | All relevant patient in-formation was available to me at the required time. | **1**  **2**  **3**  **4**  **5**  **6**  **7**  **NA** |  | 2 (1.2)  5 (2.9)  5 (2.9)  11 (6.4)  25 (14.5)  33 (19.2)  91 (52.9)  0 (0) | 3 (1.8)  1 (0.6)  6 (3.6)  12 (7.2)  30 (18)  26 (15.6)  89 (53.3)  0 (0) | 6 (21.4)  3 (10.7)  0 (0)  3 (10.7)  8 (28.6)  3 (10.7)  5 (17.9)  0 (0) |
| **Q3** |  | The in-formation on the further procedure was available to me at the required time. | The in-formation on the further procedure was available to me at the required time. | The option of tele-medical support helps me to manage the resources in my practice | **1**  **2**  **3**  **4**  **5**  **6**  **7**  **NA** |  | 5 (2.9)  3 (1.7)  5 (2.9)  9 (5.2)  15 (8.7)  51 (29.7)  84 (48.8)  0 (0) | 2 (1.2)  2 (1.2)  3 (1.8)  14 (8.4)  28 (16.8)  28 (16.8)  90 (53.9)  0 (0) | 8 (28.6)  3 (10.7)  4 (14.3)  4 (14.3)  8 (28.6)  1 (3.6)  0 (0)  0 (0) |
| **Q4** |  | The use of the exchange platform (referrer portal USB) was easy. | The use of the exchange platform (referrer portal USB) was easy. |  | **1**  **2**  **3**  **4**  **5**  **6**  **7**  **NA** |  | 17 (7.3)  0 (0)  0 (0)  2 (0.9)  1 (0.4)  5 (2.1)  9 (3.8)  200 (85.5) | 17 (54.8)  0 (0)  1 (3.2)  0 (0)  1 (3.2)  1 (3.2)  11 (35.5)  203 (86.8) |  |
| **Q5** | Compared to the usual transfer home, tele-medical support adds value for this patient. | Compared to the usual transfer home, tele-medical support adds value for this patient. | Compared to the usual transfer home, tele-medical support adds value for this patient. | Compared to the usual transfer home, tele-medical monitoring adds value for this patient. | **1**  **2**  **3**  **4**  **5**  **6**  **7**  **NA** | 4 (3)  3 (2.2)  9 (6.7)  17 (12.6)  40 (29.6)  44 (32.6)  18 (13.3)  0 (0) | 6 (3.5)  3 (1.7)  4 (2.3)  15 (8.7)  29 (16.9)  38 (22.1)  77 (44.8)  0 (0) | 9 (5.4)  8 (4.8)  4 (2.4)  17 (10.2)  20 (12)  28 (16.8)  81 (48.5)  0 (0) | 7 (25)  5 (17.9)  3 (10.7)  5 (17.9)  5 (17.9)  2 (7.1)  1 (3.6)  0 (0) |
|  | **Trust** | | | | | | | | |
| **Questions** | **Hospital physician** | **TP**  **(Day 01)** | **TP**  **(Day X0)** | **GP** | **Score, n (%)** | **Hospital physician** | **TP**  **(Day 01)** | **TP**  **(Day X0)** | **GP** |
| **Q1** | The offer of tele-medical support makes it easier to decide whether to let patients go home. | I have received clear and reliable in-formation from my medical team for the optimal further support of the patient. | I have received clear and reliable in-formation from my medical team for the optimal further support of the patient. | I received clear and reliable in-formation from my medical team for the optimal further support of the patient. | **1**  **2**  **3**  **4**  **5**  **6**  **7**  **NA** | 17 (12.6)  21 (15.6)  12 (8.9)  28 (20.7)  29 (21.5)  20 (14.8)  8 (5.9)  0 (0) | 7 (4.1)  2 (1.2)  3 (1.7)  11 (6.4)  29 (16.9)  42 (24.4)  78 (45.4)  0 (0) | 12 (7.2)  2 (1.2)  4 (2.4)  10 (6)  27 (16.2)  31 (18.6)  81 (48.5)  0 (0) | 6 (21.4)  3 (10.7)  4 (14.3)  5 (17.9)  7 (25.0)  2 (7.1)  1 (3.6)  0 (0) |
| **Q2** |  |  |  | I will re-commend tele-medical support to other suitable patients in the future. | **1**  **2**  **3**  **4**  **5**  **6**  **7**  **NA** |  |  |  | 8 (28.6)  3 (10.7)  2 (7.1)  4 (14.3)  7 (25)  3 (10.7)  1 (3.6)  0 (0) |
|  | **Empowerment** | | | | | | | | |
| **Questions** | **Hospital physician** | **TP**  **(Day 01)** | **TP**  **(Day X0)** | **GP** | **Score, n (%)** | **Hospital physician** | **TP**  **(Day 01)** | **TP**  **(Day X0)** | **GP** |
| **Q1** | In my view, tele-medical support helps the patient to organize their daily life indepen-dently after inpatient treatment. | In my view, tele-medical support helps patients to shape their everyday lives indepen-dently | In my view, tele-medical support helps patients to shape their everyday lives indepen-dently. | In my opinion, tele-medical support helps patients to live their daily lives indepen-dently. | **1**  **2**  **3**  **4**  **5**  **6**  **7**  **NA** | 4 (3)  7 (5.2)  9 (6.7)  27 (20)  43 (31.9)  33 (24.4)  12 (8.9)  0 (0) | 2 (1.2)  3 (1.7)  1 (0.6)  11 (6.4)  28 (16.3)  38 (22.1)  89 (51.7)  0 (0) | 2 (1)  5 (3)  5 (3)  15 (9)  19 (11.4)  29 (17.4)  92 (55.1)  0 (0) | 5 (17.9)  2 (7.1)  3 (10.7)  8 (28.6)  5 (17.9)  3 (10.7)  2 (7.1)  0 (0) |
|  | **Overall Satisfaction** | | | | | | | | |
| **Questions** | **Hospital physician** | **TP**  **(Day 01)** | **TP**  **(Day X0)** | **GP** | **Score, n (%)** | **Hospital physician** | **TP**  **(Day 01)** | **TP**  **(Day X0)** | **GP** |
| **Q1** | I am satisfied with the tele-medical support offered. | I am satisfied with the tele-medical support service overall. | I am satisfied with the tele-medical support service overall. | I am satisfied with the tele-medical support offered. | **1**  **2**  **3**  **4**  **5**  **6**  **7**  **NA** | 0 (0)  1 (0.7)  6 (4.4)  23 (17)  26 (19.3)  55 (40.7)  24 (17.8)  0 (0) | 0 (0.0)  0 (0.0)  1 (0.6)  11 (6.4)  24 (14.0)  44 (25.6)  92 (53.5)  0 (0) | 0 (0)  0 (0)  1 (0.6)  17 (10.2)  21 (12.6)  42 (25.2)  86 (51.5)  0 (0) | 4 (14.3)  3 (10.7)  3 (10.7)  7 (25)  7 (25)  3 (10.7)  1 (3.6)  0 (0) |

*^a^Questions across all items were rated on a seven-point Likert scale (1 = lowest, 7 = highest).*
