# Supplementary material for: Evaluating a Telemedical Follow-Up Program for Continuity of Care After Hospital Discharge: Prospective Clinical Intervention Study
Source: JMIR Form Res. 2026 Mar 27;10:e85467. doi: 10.2196/85467 (PMC13026428; doi:10.2196/85467)
Supplement: Multimedia Appendix 3 [file formative-v10-e85467-s003.docx]

**Questionnaires**

***PROM_PAT_DAY_00_01_X+2***

Mobility/agility: How would you rate your health today in terms of your mobility/agility?

- I have no problems walking around.
- I have slight problems walking around.
- I have moderate problems walking around.
- I have major problems walking around.
- I am unable to walk around.

Caring for yourself: How would you rate your health today in terms of your self-care?

- I have no problems washing or dressing myself.
- I have slight problems washing or dressing myself.
- I have moderate problems washing or dressing myself.
- I have major problems washing or dressing myself.
- I am unable to wash or dress myself.

General activities: How would you rate your health today in relation to your everyday activities? For example, work, study, housework, family, or leisure activities.

- I have no problems carrying out my everyday activities.
- I have slight problems carrying out my everyday activities.
- I have moderate problems carrying out my everyday activities.
- I have major problems going about my daily activities.
- I am unable to go about my daily activities.

Pain/physical discomfort: How would you rate your health today in terms of pain/physical discomfort?

- I have no pain or discomfort.
- I have slight pain or discomfort.
- I have moderate pain or discomfort.
- I have severe pain or discomfort.
- I have extreme pain or discomfort.

Anxiety/depression: How would you rate your health today in terms of anxiety/depression?

- I am not anxious or depressed.
- I am a little anxious or depressed.
- I am moderately anxious or depressed.
- I am very anxious or depressed.
- I am extremely anxious or depressed.

Your health today: We want to find out how good or bad your health is today.

This scale is marked with numbers from 0 to 100. 100 is the best health you can imagine. 0 (zero) is the worst health you can imagine.

***PREM_PAT_DAY_00***

The next part of the questionnaire is about your previous experience with telemedical support. There are no right or wrong answers. We are only interested in your own opinion.

Some questions are formulated as statements. You have seven possible answers to each one.

1: You strongly disagree with this statement.

2: You disagree with this statement.

3: You tend to disagree with this statement.

4: You neither agree nor disagree with this statement.

5: You somewhat agree with this statement.

6: You mostly agree with this statement

7: You strongly agree with this statement.

Is this your first inpatient hospital stay?

- Yes
- No

I feel medically well cared for with telemedical support for the transfer to home. How strongly do you agree with this statement? 1 stands for no agreement at all, 7 for a very high agreement.

What are the reasons for this? (optional)

I find the transfer process from hospital to telemedical care easy. How strongly do you agree with this statement? 1 stands for no agreement and 7 for very high agreement.

What could be improved in the transfer process? (optional)

I slept well during the inpatient stay. How strongly do you agree with this statement? 1 stands for no agreement and 7 for very high agreement.

Compared to what I have experienced with others when I was transferred home, telemedical support adds value for me. To what extent do you agree with this statement? 1 stands for no agreement and 7 for very high agreement.

Compared to being transferred home from previous hospital stays, telemedical monitoring adds value for me. How strongly do you agree with this statement? 1 stands for no agreement and 7 for very strong agreement.

What are the reasons for this? (optional)

I know what the next steps in the telemedical support are. How strongly do you agree with this statement? 1 stands for no agreement and 7 for a very high agreement.

What additional information would you have liked? (optional)

With the help of telemedical support, I will be able to shape my everyday life independently. How strongly do you agree with this statement? 1 stands for no agreement and 7 for a very high agreement.

What are the reasons for this? (optional)

Which means of transportation do you use to get to doctor's appointments? Select all that apply.

- On foot
- Bicycle
- public transportation (tram, bus, train, etc.)
- car (including taxi or other driving services)
- motorcycle or similar
- other

I think that telemedical support is a good offer. How much do you agree with this statement? 1 stands for no agreement at all, 7 for a very high agreement.

Do you have any further comments or feedback? How do you feel about the new telemedical support service so far? (optional)

Did you complete the questionnaire alone?

- alone
- with the support of relatives
- with the support of nursing staff with other support

***PREM_PAT_DAY_01***

The next part of the questionnaire is about your previous experience with telemedical support. There are no right or wrong answers. We are only interested in your own opinion. Some questions are formulated as statements. You have seven possible answers to each one.

1: You strongly disagree with this statement.

2: You disagree with this statement.

3: You tend to disagree with this statement.

4: You neither agree nor disagree with this statement.

5: You somewhat agree with this statement.

6: You mostly agree with this statement

7: You strongly agree with this statement.

I feel medically safe with the telemedical support. How strongly do you agree with this statement? 1 stands for no agreement at all, 7 for a very high agreement.

What are the reasons for this? (optional)

I find the transfer process from hospital to telemedical care easy. How strongly do you agree with this statement? 1 stands for no agreement at all, 7 for a very high level of agreement.

I found the information regarding my treatment from all medical professionals involved (hospital, telemedical support, primary care physician) to be consistent. How strongly do you agree with this statement? 1 stands for no agreement at all, 7 for very high agreement.

Telemedical monitoring made the transition from hospital to home easier for me. How much do you agree with this statement? 1 stands for no agreement and 7 for very high agreement.

What brought you relief? What could still be optimized from your point of view? (optional)

I slept well at home last night. How strongly do you agree with this statement? 1 stands for no agreement at all, 7 for a very high agreement.

Compared to what I have experienced with others when I was transferred home, telemedical support adds value for me. To what extent do you agree with this statement? 1 stands for no agreement and 7 for very high agreement.

Compared to being transferred home from previous hospital stays, telemedical monitoring adds value for me. How strongly do you agree with this statement? 1 stands for no agreement and 7 for very strong agreement.

What are the reasons for this? (optional)

I feel well looked after by the telemedical support. How strongly do you agree with this statement? 1 stands for no agreement and 7 for very high agreement.

What are the reasons for this? (optional)

I know what the next steps are in the telemedical support. How strongly do you agree with this statement? 1 stands for no agreement at all, 7 for very high agreement.

I know who I can turn to if I have any questions. How strongly do you agree with this statement? 1 stands for no agreement and 7 for a very high level of agreement.

With the help of telemedical support, I can shape my everyday life independently. How much do you agree with this statement? 1 stands for no agreement and 7 for a very high level of agreement.

What are the reasons for this? (optional)

I am satisfied with the telemedical support offered. How much do you agree with this statement? 1 stands for no agreement and 7 for a very high agreement.

Do you have any further comments or feedback? How do you feel so far about the new telemedical support service? (optional)

What feedback have you received from your relatives about the telemedical support? (optional)

Did you complete the questionnaire alone?

- alone
- with the support of relatives
- with the support of nursing staff
- with other support¨

***PREM_PAT_DAY_X+2***

The next part of the questionnaire is about your previous experience with telemedical support. There are no right or wrong answers. We are only interested in your own opinion. Some questions are formulated as statements. You have seven possible answers to each one.

1: You strongly disagree with this statement.

2: You disagree with this statement.

3: You tend to disagree with this statement.

4: You neither agree nor disagree with this statement.

5: You somewhat agree with this statement.

6: You mostly agree with this statement

7: You strongly agree with this statement.

I felt medically safe with the telemedical support. How strongly do you agree with this statement? 1 stands for no agreement at all, 7 for a very high level of agreement.

What are the reasons for this? (optional)

I find the transfer process from hospital to telemedical support easy. How strongly do you agree with this statement? 1 stands for no agreement at all, 7 for a very high level of agreement.

I found the information regarding my treatment from all medical professionals involved (hospital, telemedical support, primary care physician) to be consistent. How strongly do you agree with this statement? 1 stands for no agreement at all, 7 for very high agreement.

Telemedical monitoring made the transition from hospital to home easier for me. How much do you agree with this statement? 1 stands for no agreement and 7 for very high agreement.

What helped you feel better? What could still be improved in your view? (optional)

I slept well at home the last few nights. How much do you agree with this statement? 1 stands for no agreement and 7 for very strong agreement.

Compared to what I have experienced with others when transferring home after previous hospital stays, telemedical support adds value for me. How strongly do you agree with this statement? 1 stands for no agreement and 7 for very high agreement.

Compared to being transferred home from previous hospital stays, telemedical monitoring adds value for me. How much do you agree with this statement? 1 stands for no agreement and 7 for very high agreement.

What are the reasons for this? (optional)

I felt well looked after by the telemedical support. How strongly do you agree with this statement? 1 stands for no agreement and 7 for very high agreement.

What are the reasons for this? (optional)

With the help of telemedical support, I was able to shape my everyday life independently. How strongly do you agree with this statement? 1 stands for no agreement and 7 for very high agreement.

What are the reasons for this? (optional)

I am satisfied with the telemedical support offered. How much do you agree with this statement? 1 stands for no agreement and 7 for very high agreement.

Now think about your experience with telemedical support when completing the following questions, in particular your interaction with the Medgate app. These are pairs of contrasting characteristics. For each pair of words, assess which characteristic best matches your experience.

Which characteristic of this pair of words best matches your experience?

- hindering
- supporting

Which of these best describes your experience?

- Complicated
- Simple

Which of these best describes your experience?

- Confusing
- Clear

Which of these best describes your experience?

- Boring
- Exciting

Which of these best describes your experience?

- Uninteresting
- Interesting

Which of these words best describes your experience?

- Conventional
- Original

Which of these words best describes your experience?

- Usual
- Novel

Do you have any further comments or feedback? How do you feel about the new telemedical support service so far? (optional)

What feedback do you have from your relatives about the telemedical support? (optional)

Did you complete the questionnaire alone?

- Alone
- with the support of relatives
- with the support of nursing staff
- with other support

We would like to continuously improve the telemedical support service. Would you be willing to take part in an additional telephone interview (approx. 10 minutes) as part of the ongoing pilot project?

- Yes
- No

Please provide contact details on how we can best reach you (telephone or email, if applicable, ideal time frame)

***PREM_USB_DAY_00***

This questionnaire is about your previous experience with the telemedical support program. It will take about 10 minutes to complete the questionnaire. There are no right or wrong answers. We are only interested in your own opinion.

Consent I have been informed about the study and agree to participate in this study.

- I agree

Some questions are formulated as statements. You have seven possible answers to each one.

1: You strongly disagree with this statement.

2: You disagree with this statement.

3: You tend to disagree with this statement.

4: You neither agree nor disagree with this statement.

5: You somewhat agree with this statement.

6: You mostly agree with this statement

7: You strongly agree with this statement.

I feel that this patient is suitable for telemedical monitoring. How strongly do you agree with this statement? 1 stands for no agreement at all, 7 for a very high level of agreement.

What are the reasons for this? (optional)

I find the process of transferring this patient from the hospital to telemedical care easy. How strongly do you agree with this statement? 1 stands for no agreement at all, 7 for a very high agreement.

What could possibly be improved in the transfer process? (optional)

Compared to the usual transfer home, telemedical support adds value for this patient. To what extent do you agree with this statement? 1 stands for no agreement and 7 for very high agreement.

What are the reasons for this? What do you find better or worse? (optional)

The offer of telemedical support makes it easier to decide whether to let patients go home. To what extent do you agree with this statement? 1 stands for no agreement and 7 for a very high level of agreement.

What are the reasons for this? (optional)

Thanks to the offer of telemedical support, this patient was able to go home earlier.

- Yes
- No

How many days longer would you have kept this patient in the hospital without the offer of telemedical support?

- 1 day longer stay
- 2 days longer stay
- more than 2 days longer stay

From my point of view, telemedical support helps the patient to organize their daily life independently after inpatient treatment. To what extent do you agree with this statement? 1 stands for no agreement and 7 for a very high agreement.

What are the reasons for this? (optional)

I am satisfied with the telemedical support offered. How strongly do you agree with this statement? 1 stands for no agreement and 7 for very high agreement.

Do you have any further comments or feedback? How do you feel so far about the new telemedical support service? (optional)

***PREM_MG_DAY_01***

This questionnaire is about your previous experience with the telemedical support service. It will take about 10 minutes to complete the questionnaire. There are no right or wrong answers. We are only interested in your own opinion.

Consent I have been informed about the study and agree to participate in this study.

- I agree

Some questions are formulated as statements. You have seven possible answers for each question.

1: You strongly disagree with this statement.

2: You disagree with this statement.

3: You tend to disagree with this statement.

4: You neither agree nor disagree with this statement.

5: You somewhat agree with this statement.

6: You mostly agree with this statement

7: You strongly agree with this statement.

I feel that this patient is suitable for telemedical support. How strongly do you agree with this statement? 1 represents no agreement at all, 7 represents a very high level of agreement.

What are the reasons for this? (optional)

I find the process of transferring this patient from hospital to telemedical care simple. How strongly do you agree with this statement? 1 stands for no agreement and 7 for a very high level of agreement.

What could be improved in the transfer process? (optional)

All relevant patient information was available to me at the required time. How strongly do you agree with this statement? 1 stands for no agreement, 7 for a very high agreement.

The information on the further procedure was available to me at the required time. How strongly do you agree with this statement? 1 stands for no agreement, 7 for a very high agreement.

What additional information would you have liked? What could be improved in the information transfer? (optional)

The use of the exchange platform (referrer portal USB) was easy. How much do you agree with this statement? 1 stands for no agreement, 7 for a very high agreement.

What could possibly be improved? (optional)

Compared to the usual transfer home, telemedical monitoring adds value for this patient. How strongly do you agree with this statement? 1 stands for no agreement and 7 for very high agreement.

What are the reasons for this? What do you find better or worse? (optional)

I have received clear and reliable information from my medical team

for the optimal further support of the patient. How strongly do you agree with this statement? 1 stands for no agreement and 7 for very high agreement.

What additional information would have been useful? (optional)

In my view, telemedical support helps patients to shape their everyday lives independently. How strongly do you agree with this statement? 1 stands for no agreement and 7 for a very high agreement.

What are the reasons for this? (optional)

I am satisfied with the telemedical support service overall. How strongly do you agree with this statement? 1 stands for no agreement and 7 for very high agreement.

Do you have any further comments or feedback? How do you feel about the new telemedical support service so far? (optional)

***PREM_MG_DAY_X0***

This questionnaire is about your previous experience with telemedical support. It should take about 10 minutes to complete. There are no right or wrong answers. We are only interested in your own opinion.

Consent: I have been informed about the study and agree to participate in this study. I agree.

How many consultations did this patient have?

How many Medgate doctors were involved in the telemedical care of this patient?

How did this patient complete the telemedical care? Study completion – transfer to GP via referral completed Study completion – rehospitalization Study completion – no GP available (transfer to other medical institution/care) Study completion – no GP available (independent ongoing care) Study termination – patient no longer wishes to participate in the study Study exclusion – non-compliance Study exclusion – other (please specify in the comments field)

Study termination: Did the patient give reasons? If so, what were they?

Study exclusion: What exactly was the reason?

When did the patient complete/terminate the study or when was the study terminated?

Some of the following questions are formulated as statements. They each have seven possible answers.

1: You strongly disagree with this statement.

2: You disagree with this statement.

3: You tend not to agree with this statement.

4: You neither agree nor disagree with this statement.

5: You somewhat agree with this statement.

6: You mostly agree with this statement

7: You strongly agree with this statement.

I feel that this patient was suitable for telemedical support. On a scale of 1 to 7, where 1 is strongly disagree and 7 is strongly agree, how much do you agree with this statement?

What are the reasons for this? (optional)

I find the process of transferring this patient from telemedical support to the family doctor straightforward. On a scale of 1 to 7, where 1 is strongly disagree and 7 is strongly agree, how much do you agree with this statement?

What could possibly be improved in the transfer process? (optional)

All relevant patient information was available to me at the required time. How strongly do you agree with this statement? 1 stands for no agreement, 7 for a very high agreement.

The information on the further procedure was available to me at the required time. How strongly do you agree with this statement? 1 stands for no agreement, 7 for a very high agreement.

What additional information would you have liked?

What could be improved when the information is provided? (optional)

Using the exchange platform (referrer portal USB) was easy. How much do you agree with this statement? 1 stands for no agreement, 7 for a very high agreement.

What could possibly be improved? (optional)

Compared to the usual transfer home, telemedical support adds value for this patient. How strongly do you agree with this statement? 1 stands for no agreement and 7 for a very high agreement.

What are the reasons for this? What do you find better or worse? (optional)

I have received clear and reliable information from my medical team for the optimal further support of the patient. How strongly do you agree with this statement? 1 stands for no agreement and 7 for a very high agreement.

What additional information would have been useful? (optional)

In my view, telemedical support helps patients to shape their everyday lives independently. How strongly do you agree with this statement? 1 stands for no agreement and 7 for a very high level of agreement.

What are the reasons for this? (optional)

I am satisfied with the telemedical support service overall. How strongly do you agree with this statement? 1 stands for no agreement and 7 for a very high level of agreement.

Do you have any further comments or feedback? How do you feel about the new telemedical support service so far? (optional)

***PREM_HA_DAY_X+2***

This questionnaire is about your previous experience with telemedical support. It will take about 10 minutes to complete the questionnaire. There are no right or wrong answers. We are only interested in your own opinion.

Consent I have been informed about the study and agree to participate in this study.

- I agree

Have you had contact with this patient since the telemedical support?

- No
- Yes, for a general check-up
- Yes, for an emergency
- Yes, for another reason

What was the reason?

Some questions are formulated as statements. You have seven possible answers in each case.

1: You strongly disagree with this statement.

2: You disagree with this statement.

3: You tend to disagree with this statement.

4: You neither agree nor disagree with this statement.

5: You somewhat agree with this statement.

6: You mostly agree with this statement

7: You strongly agree with this statement.

I feel that this patient was suitable for telemedical support. How strongly do you agree with this statement? 1 stands for no agreement at all, 7 for a very high level of agreement.

What are the reasons for this? (optional)

I find the transfer process from telemedical support to the family doctor easy. How strongly do you agree with this statement? 1 stands for no agreement and 7 for very high agreement.

What could be improved in the transfer process, if anything? (optional)

All relevant patient information was available to me at the required time. How strongly do you agree with this statement? 1 stands for no agreement and 7 for a very high level of agreement.

What additional information would you have liked? What can be improved in the information transfer? (optional)

The option of telemedical support helps me to manage the resources in my practice. How much do you agree with this statement? 1 stands for no agreement and 7 for a very high agreement.

Compared to the usual transfer home, telemedical support adds value for this patient. How much do you agree with this statement? 1 stands for no agreement and 7 for very high agreement.

What are the reasons for this? What do you like or dislike about this? (optional)

I received clear and reliable information from my medical team for the optimal further support of the patient. How much do you agree with this statement? 1 stands for no agreement and 7 for a very high agreement.

What additional information would have been useful? (optional)

I will recommend telemedical support to other suitable patients in the future. How much do you agree with this statement? 1 stands for no agreement and 7 for a very high agreement.

What are the reasons for this? (optional)

In my opinion, telemedical support helps patients to live their daily lives independently. To what extent do you agree with this statement? 1 represents no agreement and 7 represents a very high level of agreement.

What are the reasons for this? (optional)

I am satisfied with the telemedical support offered. How strongly do you agree with this statement? 1 stands for no agreement and 7 for very high agreement.

Do you have any further comments or feedback? How do you feel about the new telemedical support service so far? (optional)

We would like to continuously improve the telemedical support service. Would you be willing to take part in an additional telephone interview (approx. 10 minutes) as part of the ongoing study?

- Yes
- No

Please provide contact details on how we can best reach you (telephone or email, if applicable, ideal time frame)
